# Epidemiology and clinical course of COVID-19 in Shanghai, China

**DOI:** 10.1080/22221751.2020.1787103

**Published:** 2020-07-07

**Authors:** Yinzhong Shen, Fang Zheng, Danfeng Sun, Yun Ling, Jun Chen, Feng Li, Tao Li, Zhiping Qian, Yuyi Zhang, Qingnian Xu, Li Liu, Qin Huang, Fei Shan, Lie Xu, Jun Wu, Zhaoqin Zhu, Zhigang Song, Shenyang Li, Yuxin Shi, Jianliang Zhang, Xueyun Wu, Joshua B. Mendelsohn, Tongyu Zhu, Hongzhou Lu

**Affiliations:** a Department of Infection and Immunity, Shanghai Public Health Clinical Center, Fudan University, Shanghai, People’s Republic of China; b Department of Medical Affairs, Shanghai Public Health Clinical Center, Fudan University, Shanghai, People’s Republic of China; c Department of Infection, Shanghai Public Health Clinical Center, Fudan University, Shanghai, People’s Republic of China; d Department of Respiratory Medicine, Shanghai Public Health Clinical Center, Fudan University, Shanghai, People’s Republic of China; e Department of Tuberculosis, Shanghai Public Health Clinical Center, Fudan University, Shanghai, People’s Republic of China; f Department of Severe Liver Disease, Shanghai Public Health Clinical Center, Fudan University, Shanghai, China; g Department of Traditional Chinese Medicine, Shanghai Public Health Clinical Center, Fudan University, Shanghai, People’s Republic of China; h Department of Radiology, Shanghai Public Health Clinical Center, Fudan University, Shanghai, People’s Republic of China; i Clinical Laboratory, Shanghai Public Health Clinical Center, Fudan University, Shanghai, People’s Republic of China; j P3 Laboratory, Shanghai Public Health Clinical Center, Fudan University, Shanghai, People’s Republic of China; k Data Management Center, Shanghai Public Health Clinical Center, Fudan University, Shanghai, People’s Republic of China; l Department of Gastroenterology, Shanghai Public Health Clinical Center, Fudan University, Shanghai, People’s Republic of China; m College of Health Professions, Pace University, New York, NY, USA; n Department of Urology, Shanghai Public Health Clinical Center, Fudan University, Shanghai, People’s Republic of China

**Keywords:** COVID-19, epidemiology, clinical characteristics, clinical course, viral shedding

## Abstract

**Background:** Novel coronavirus pneumonia (COVID-19) is prevalent around the world. We aimed to describe epidemiological features and clinical course in Shanghai.

**Methods:** We retrospectively analysed 325 cases admitted at Shanghai Public Health Clinical Center, between January 20 and February 29, 2020.

**Results:** 47.4% (154/325) had visited Wuhan within 2 weeks of illness onset. 57.2% occurred in 67 clusters; 40% were situated within 53 family clusters. 83.7% developed fever during the disease course. Median times from onset to first medical care, hospitalization and negative detection of nucleic acid by nasopharyngeal swab were 1, 4 and 8 days. Patients with mild disease using glucocorticoid tended to have longer viral shedding in blood and feces. At admission, 69.8% presented with lymphopenia and 38.8% had elevated D-dimers. Pneumonia was identified in 97.5% (314/322) of cases by chest CT scan. Severe-critical patients were 8% with a median time from onset to critical disease of 10.5 days. Half required oxygen therapy and 7.1% high-flow nasal oxygen. The case fatality rate was 0.92% with median time from onset to death of 16 days.

**Conclusion:** COVID-19 cases in Shanghai were imported. Rapid identification, and effective control measures helped to contain the outbreak and prevent community transmission.

## Introduction

The novel coronavirus SARS-CoV-2 was not known to infect humans until very recently [1]. It was declared a global public health emergency of international concern on January 30 and a pandemic on March 11, 2020, the first to be caused by a coronavirus [2,3]. COVID-19 is an emerging infectious disease that has exerted a tremendous impact on public health and socioeconomic development. Confirmed cases have been reported in 196 countries, areas or territories [4]. As of March 25, 2020, 375,498 cases including 16,362 deaths were reported worldwide [4]. The first case was reported in Shanghai on January 20, 2020. By February 29, of 337 cases confirmed in Shanghai, 287 were discharged and 3 died. While COVID-19 cases in Shanghai have all been imported, we are not aware of a comprehensive analysis of the epidemiological and clinical course of primarily imported COVID-19 in a major urban centre. The Shanghai Public Health Clinical Center (SPHCC) is the only COVID-19 designated hospital for adult patients in Shanghai. We investigated epidemiological and clinical features, disease severity, diagnosis, treatment, clinical outcomes, and follow-up of COVID-19 in Shanghai, with the hope of assisting other large urban centres in planning for the high risk of extensive SARS-CoV-2 transmission. The effective management of this epidemic highlights the benefits of early and aggressive control measures.

## Methods

### Subjects

COVID-19 confirmed patients admitted in the Emergency Response Department of SPHCC January 20-February 29 were included. Epidemiological history, clinical manifestations, laboratory test results, and imaging test results were retrospectively collected from medical records. The study was approved by SPHCC Ethics Committee.

### Diagnosis and admission process

Individuals typically presented at Shanghai’s district hospitals (including SPHCC). Real-time reverse transcriptase polymerase chain reaction (RT–PCR) assay for confirming SARS-CoV-2 infection were conducted in local Centres for Disease Control and Prevention (CDC) according to WHO protocols [5]. Patients with positive nucleic acid test results were transferred to SPHCC for further treatment using negative pressure ambulances.

### Follow-up after discharge

Patients were followed-up at 2- and 4-weeks post-discharge to ascertain if any contacts had respiratory symptoms or fever. Blood counts, liver and kidney function, and nucleic acid tests were completed on oro- or nasopharyngeal swabs or in sputum. Chest computed tomography (CT) was provided as needed.

### Related definitions

Diagnoses of COVID-19 were carried out according to national guidelines [6]. Severe cases were defined as dyspnoea, respiratory frequency ≥30/min, blood oxygen saturation ≤93%, partial pressure of arterial oxygen to fraction of inspired oxygen ratio <300 mmHg, and/or lung infiltrates >50% within 24–48 h. Critical cases were defined as having respiratory failure, septic shock, and/or multiple organ dysfunction or failure. Clinical outcomes, including continued hospitalization, discharge and death, were recorded as of February 29, 2020.

### Statistical analysis and plotting

Measurement data were expressed as mean ± standard deviation or median with interquartile range. Enumeration data were expressed as percentages and rates. T-tests and non-parametric tests were used to compare continuous data. *Chi*-square tests were used to compare counts. The level of significance was set at 0.05. SPSS 20.0 was used to analyse the data. All figures were created with Microsoft PowerPoint 2016.

## Results

### Epidemiological features

**Figure 1.**
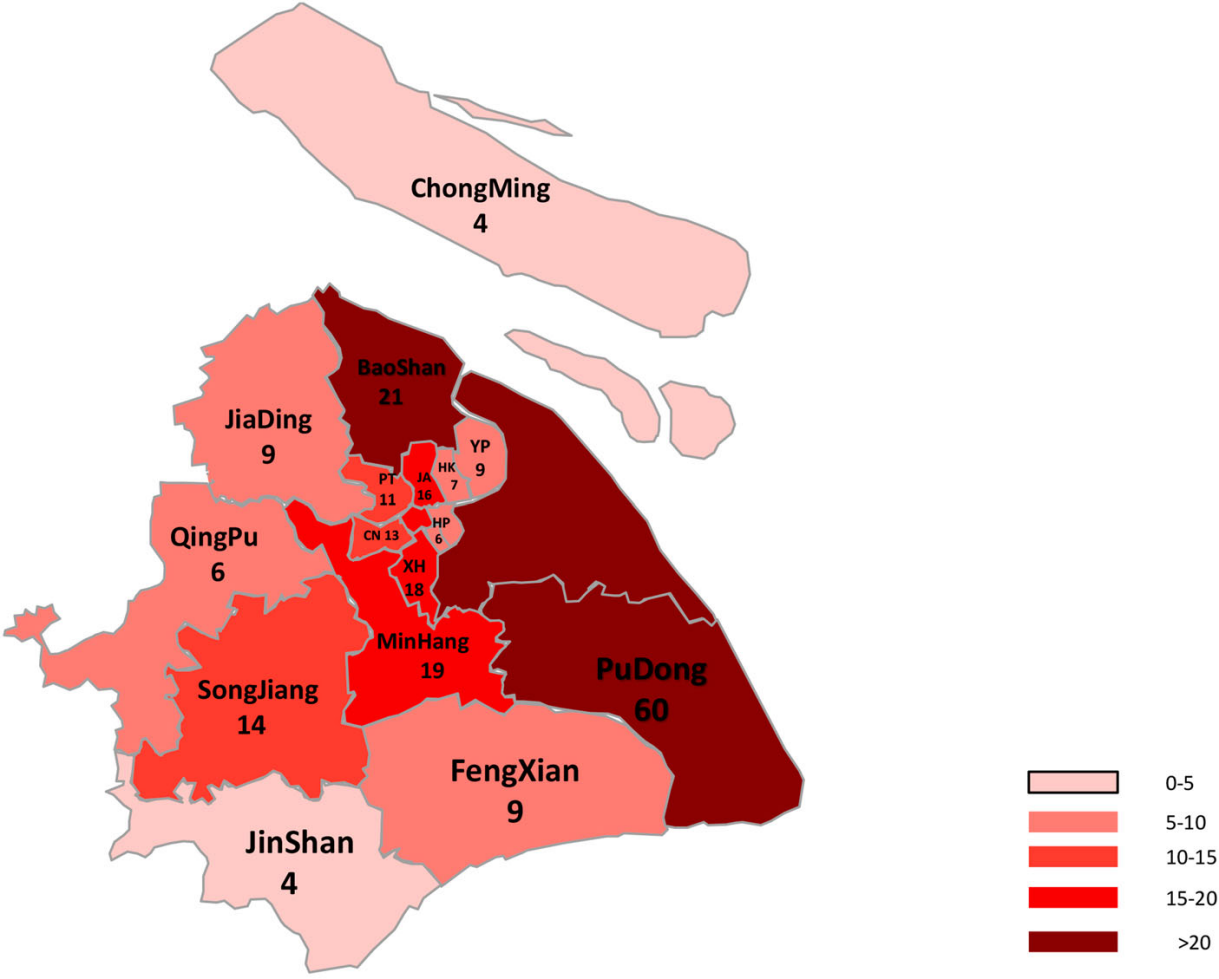
The geographic distribution of Shanghai residents admitted to Shanghai Public Health Clinical Center. YP: Yangpu District; HK: Hongkou District; JA: Jing’an District; HP: Huangpu District; PT: Putuo District; CN: Changning District; XH: Xuhui District. Numbers indicate the total cases occurred in the corresponding district. Residents from other cities are not included.

**Figure 2.**
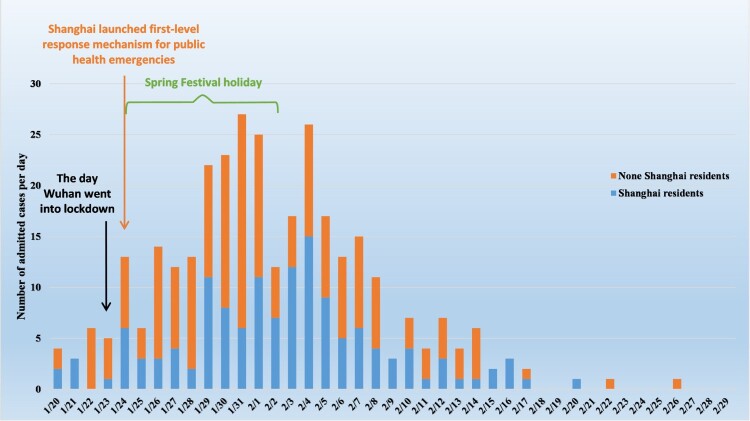
Daily distribution of confirmed cases of COVID-19 in Shanghai Public Health Clinical Center from January 20 to February 29, 2020.

**Figure 3.**
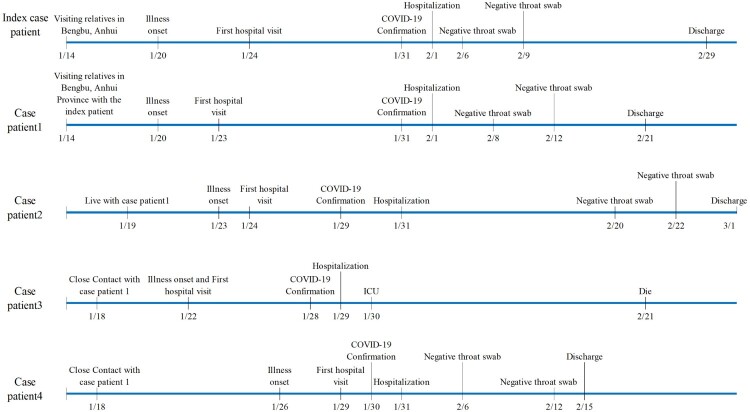
Pathogen exposures and clinical courses in a clustered event.

Drawing from all 16 districts of Shanghai (Figure 1), 325 confirmed COVID-19 cases were admitted to the Emergency Response Department of SPHCC, January 20-February 29 (Figure 2). Cases peaked January 29-February 5, with half (52%) admitted during this period. Median age was 51 years (range, 15–88; IQR, 36–64), 20% (65/325) were over 65 years old, and 52% (168/325) were male (Figure S1 in supplementary material). More than half of cases (57.2%; 186/325) were residents in other provinces. In the 14 days prior to disease onset, almost half (47.4%; 154/325) had visited Wuhan (the capital city of Hubei province, 800 kilometres west of Shanghai), while just over a third (35.4%; 115/325) reported contact with a confirmed case (Table 1). Of 67 cluster events involving 186 (57.2%) cases, 53 (79%) were family clusters comprising 40.3% (131/325) of all cases, 3 were colleague-clustered events, and 3 were family-colleague mixed clustered events (Table 1). Typical cluster event features are shown in Figure 3.

**Table 1.** Epidemiologic characteristics of 325 patients with COVID-19 in Shanghai, China.

Characteristic	Value
Age—year	
Mean	50
Median	51
Interquartile range	36–64
Age ≤17 years— no. (%)	3 (0.92)
18 years ≤ Age ≤40 years— no. (%)	116 (35.7)
41years ≤ Age ≤65 years— no. (%)	141 (43.4)
Age≥66 years— no. (%)	65 (20.0)
Male sex— no. (%)	168 (51.7)
Occupational classification— no. (%)	
Retired personnel	130 (40)
Cadre staff	111 (34.2)
Workers	42 (12.3)
Classification of clinical severity— no. (%)	
Mild	299 (92)
Severe	10 (3.1)
Critical	16 (4.9)
Type of residence — no. (%)	
Shanghai	139 (42.8)
Other provinces except Shanghai	186 (57.23)
History of visiting within 14 days before the onset of illness — no./total no. (%)	205/325 (63.1)
Wuhan	154/325 (47.4)
Outside Wuhan in Hubei	24/325 (7.4)
Provinces other than Hubei	27/325 (8.3)
Cluster case — no./total no. (%)	186/325 (57.2)
Family clusters (53)	131/325 (40.3)
Colleague clusters (3)	7/325 (2.2)
Family-colleague mixing clusters (3)	21/325 (6.5)
Other clusters (8)	27/325 (8.3)
Length of hospital stay—day	
Mean	15.4
Median	15
Interquartile range	11–20
Outcome — no./total no. (%)	
Discharged	279/325 (85.8)
Currently hospitalized	43/325 (13.2)
Death	3/325 (0.92)

### Clinical manifestations

Among all cases, 77.2% (251/325) had fever at illness onset, 16.0% (52/325) had fever ever during hospitalization, and 6.8% (22/325) never developed fever. Fever occurred in 83.7% (272/325) of hospitalized cases. Cough at onset was reported by 28.9% (94/325) of cases. Underlying co-morbidities were present in 28.9% (94/325) of cases, included hypertension (15.6%; 51/325), diabetes (7.1%; 23/325), and coronary heart disease (3.4%; 11/325). Twenty patients (6.2%) had hepatitis B surface antigens (HBsAg), three had hepatitis C antibodies, and one was co-infected with human immunodeficiency virus.

Median interval times from illness onset to: first medical care was 1 d (IQR, 0–3 days); to admission was 4 days (IQR, 2–7.5 days); to confirmed diagnosis was 4 days (IQR, 2–7 days); to positive nucleic acid result was 4 days (IQR, 2–7 days). Median interval from first medical care to positive nucleic acid result was 2 days (IQR, 1–4 days).

At admission, over two-thirds (69.8%; 227/325) presented with lymphopenia; 67.1% (218/325) with an elevated blood glucose level. Over half (56.5%; 182/322) had increased C-reactive protein (CRP); 38.8% (126/325) had elevated D-dimers; a quarter had leukocytopenia (25.2%; 82/325) or raised procalcitonin (PCT) (25.6%; 84/325); and 17.2% (56/325) had hypokalemia. Patients with CD4^+^ T-lymphocyte count <200/mm^3^ and <500/mm^3^ accounted for 10.2% (33/325) and 58.8% (191/325) of cases, respectively (Table 2). In chest CT scans, most (97.5%; 314/322) revealed typical pneumonia and ground-glass opacification (93.5%, 301/322), and less than half (42.9%, 138/322) had bilateral lung involvement. Pleural thickening was found in 64.9% (209/322) of scans.

**Table 2.** Laboratory results of 325 patients with COVID-19 in Shanghai, China.

Variable	Value-no./total no. (%)
Leukocyte (per mm^3^) <4,000	82/325 (25.2)
Lymphocyte (per mm3) <1500	227/325 (69.9)
Platelet (10^3^ per mm3) <100	12/325 (3.7)
Alanine aminotransferase (U/liter) > 40	53/325 (16.3)
Aspartate aminotransferase (U/liter) > 40	54/325 (16.6)
Creatinine(µmol/liter) > 133	0/325 (0)
Creatine kinase (U/liter) > 200	47/325 (14.46)
Serum lactate dehydrogenase(U/liter) > 250	125/325 (38.5)
Troponin I (ng/ml) > 0.04	80/325 (24.6)
B-type natriuretic peptide precursor(pg/ml) > 250	22/325 (6.8)
Myoglobin (ng/ml) > 48.8	28/325(8.6)
Potassium (mmol/liter) < 3.5	56/325(17.2)
Sodium (mmol/liter) < 135	29/325(8.9)
Glucose (mmol/liter) > 6.1	218/325(67.1)
C-reactive protein (mg/liter) > 10	182/322(56.5)
Procalcitonin(ng/ml) > 0.05	84/325(25.9)
β-glucan (pg/ml) > 60	5/13(38.5)
CD4+T lymphocyte count<200/mm^3^	33/325(10.2)
CD4+T lymphocyte count<500/mm^3^	191/325(58.8)
Fibrinogen (g/liter) > 4.0	195/325(0.6)
Fibrin degradation product (µg/ml) > 5.0	16/325(4.9)
D-dimer (µg/ml) > 0.5	126/325(38.8)

**Figure 4.**
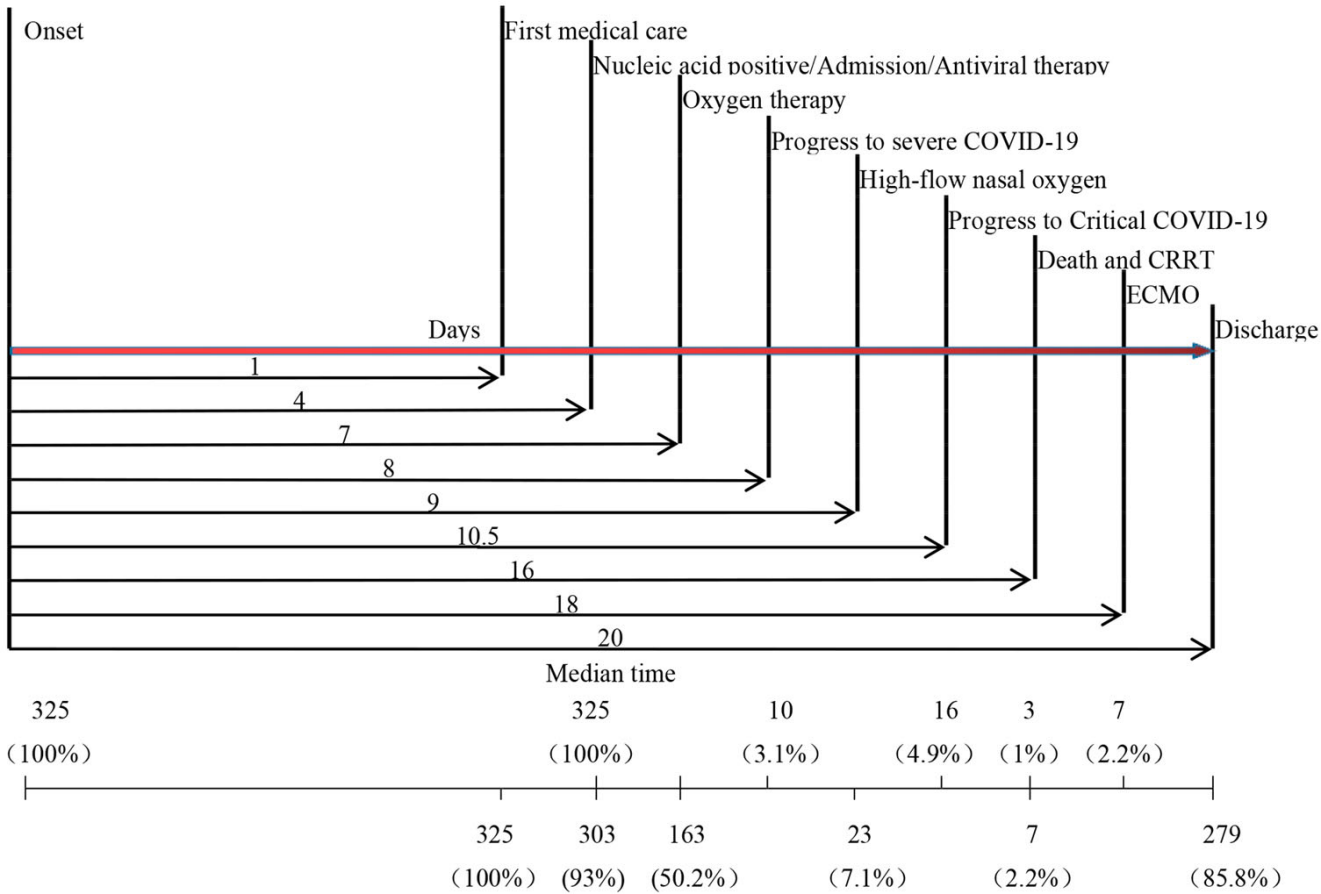
Time distribution of clinical events in COVID-19 cases admitted to Shanghai Public Health Clinical Center. Numbers underneath indicate the number of patients who went through the corresponding events. CRRT: continuous renal replacement therapy; ECOM: Extra-corporeal membrane oxygenation

The proportion of mild, severe, and critical disease was 92.0% (299/325), 3.1% (10/325), and 4.9% (16/325), respectively. The median time from onset to severe and critical illness was 8 and 10.5 days, respectively (Figure 4).

### Clinical treatment and outcomes

Half of admitted patients (50.2%; 163/325) required oxygen during the disease course with 7.1% (23/325) needing high-flow nasal oxygen (HFNO). Mechanical ventilation was required by 7.1% (23/325) with extracorporeal membrane oxygenation (ECMO) and continuous renal replacement therapy (CRRT) each given to 2.2% (7/325). Median time from illness onset to oxygen therapy, HFNO, CRRT and ECMO were 7, 9, 16, and 18 days, respectively (Figure 4). Before admission, 16.3% (53/325) had used oseltamivir, with 3 continuing the drug after admission. Only one patient was coinfected with influenza B virus.

Antiviral therapies were prescribed to 93.2% (303/325) of patients, with treatment consisting of at least one of α-interferon, lopinavir/ritonavir, and/or arbidol. Other prescribed medications included antibiotics (38.2%; 124/325), thymosin (46.2%; 150/325), anticoagulant drugs i.e. low molecular weight heparin or heparin (22.8%; 74/325), albumin (22.8%; 74/325), glucocorticoid i.e. methylprednisolone sodium succinate (15.4%; 50/325), and vasoactive drugs (5.2%; 17/325). Among mild cases, 22/299 (74%) did not receive antiviral therapy. Very few patients (1.8%; 6/325) were given convalescent plasma therapy.

Among those treated with antivirals, median times from illness onset to negative viral detection by pharyngeal swab, urine, faeces, and blood samples were 8, 8, 10, and 14 days, respectively. Median time from illness onset to negative viral detection was longer in severe and critical patients compared with mild patients (Table 3). Median times from initiation of antiviral therapies to negative viral detection in pharyngeal swab, urine, faeces, and blood samples were 2, 2, 4, and 9 days, respectively, which were longer in severe and critical patients compared with mild patients (Table 3). Among 22 mild patients without antiviral treatment, median times from illness onset to negative viral detection in nasopharyngeal swab, urine, faeces, and blood samples were 9, 7, 10, and 9.5 days, respectively. There were no differences between patients with or without antiviral treatment in time from disease onset to viral conversion (swab, *p* = 0.512; urine, *p* = 0.832; faeces, *p* = 0.401; and blood, *p* = 0.057). When limiting the comparison to mild cases only, there was no evidence for any differences across sample types (Table S1 in supplementary material). However, longer duration of viral shedding was detected in patients prescribed with glucocorticoid, especially in blood and faeces (Table S2 in supplementary material). Longer duration of viral shedding was detected in blood and faeces in patients with mild disease using glucocorticoid (Table S3 in supplementary material); however, no differences in duration of viral shedding were observed in patients with severe-critical disease (Table S4 in supplementary material).

**Table 3.** Viral shedding in COVID-19 patients with different severity.

Variable	Value			*P* Value
	Overall (*n* = 303)	Mild (*n* = 277)	Severe-critical (*n* = 26)	
Time from onset to negative nucleic acid detection in pharyngeal swab—days	8 (5–12)	8 (5–11)	16 (8–22.5)	<0.001
Time from onset to negative nucleic acid detection in blood—days	14 (10–18)	13 (10–17)	22.5 (16.5–30)	<0.001
Time from onset to negative nucleic acid detection in urine—days	8 (5–11)	7 (5–10)	12 (7–21)	<0.001
Time from onset to negative nucleic acid detection in faeces—days	10 (7–15)	10 (7–13)	21 (16.8–29)	<0.001
Time from antiviral therapy to negative nucleic acid detection in pharyngeal swab—days	2 (1–6)	2 (1–6)	11 (3.8–15.5)	<0.001
Time from antiviral therapy to negative nucleic acid detection in blood—days	9 (5–12)	9 (2–11)	19 (13.8–24)	<0.001
Time from antiviral therapy to negative nucleic acid detection in urine—days	2 (1–4)	1 (1–4)	6 (2–14.5)	<0.001
Time from antiviral therapy to negative nucleic acid detection in faeces—days	4 (2–10)	4 (1–8)	15.5 (11.5–20.8)	<0.001

Values are presented by median (interquartile range). Median values of mild and severe-critical cases are compared with Mann–Whitney *U* test.

The first discharge was on January 27; 85.8% of patients were discharged within 34 days with a median hospitalization stay of 15 days (range, 6–25 days; IQR, 6–25 days). Time from disease onset to discharge was 20 days (Table 4). There were 43 (13.2%) patients who remained under care in our hospital, one in severe and 11 in critical condition. Three deaths, all caused by acute respiratory distress syndrome (ARDS) or multiple organ dysfunction (MODS), yielded a case fatality rate of 0.92% (3/325) with median time from onset to death of 16 days (Table 4). Case fatality rate in patients with glucocorticoid therapy was 12%, while no patient died among those without glucocorticoid (Table S2).

**Table 4.** Clinical course of 325 patients with COVID-19 in Shanghai, China.

Variable	Value
Time from onset to first medical care—day	1 (0–3)
Time from onset to positive nucleic acid detection in pharyngeal swab —days	4 (2–7)
Time from first medical care to positive nucleic acid detection in pharyngeal swab —days	2 (1–4)
Time from onset to hospitalization—days	4 (2–7.5)
Time from onset to antiviral treatment—days	4 (3–7)
Time from onset to severe illness—days	8 (4–10)
Time from onset to critical illness—days	10.5 (5.3–12.5)
Time from onset to oxygen therapy—days	7 (4–9)
Time from onset to HFNO—day	9 (6–11)
Time from onset to ECMO—days	18 (10–33)
Time from onset to CRRT—days	16 (6–18)
Time from onset to death—days	16 ± 13.7*
Time from onset to discharge—days	20 (16–25)

Values are presented by median (interquartile range). *Mean value. HFNO: High-flow nasal oxygen. ECMO: Extracorporeal membrane oxygenation. CRRT: Continuous renal replacement therapy.

Some patients suffered from secondary infections. Bloodstream infections appeared in 23.5% (4/17) of patients with severe-critical illness, caused by *Staphylococcus haemolyticus* (*n* = 4), *Candida albicans* (*n* = 1), and *Cryptococcus neoformans* (*n* = 1). Half (9/17) in severe-critical condition had positive urine culture. All the 17 patients with severe-critical illness were offered with glucocorticoid. Positive rates in severe-critical cases for sputum culture, throat swab culture and stool culture were 100% (22/22), 100% (18/18) and 85.7% (6/7), respectively.

### Clinical follow-up

As of February 29, 2020, of 188 patients actively followed up one week after discharge, 31 (16.5%) reported mild discomfort and none reported fever. Mild discomfort included cough (*n* = 19), chest tightness (*n* = 4), shortness of breath (*n* = 5), night sweats (*n* = 5), and gastrointestinal discomfort (*n* = 3). Of 112 patients who attended follow-up two weeks post-discharge, there were no reported fevers, but 6 (5.4%) complained of cough and chest tightness. No patients required hospitalization at follow-up.

## Discussion

This report details the epidemiological and clinical features of COVID-19, including 325 RT–PCR confirmed cases transferred to SPHCC from all districts of Shanghai. The findings are consistent with a typical imported outbreak. Nearly half of cases had travelled to Wuhan prior to disease onset, compared with 31.3% nationally [7]. Consistent with previous studies, nearly 60% of cases were located in clusters, with two-thirds of these (40% of all cases) situated in families, implying a considerable role for household transmission [8–10]. Since effective treatment is lacking, personal preventive measures, including case isolation within households and household quarantine, were exceedingly important for prevention of onward viral transmission.

Most confirmed cases were reported between late January and early February 2020, with the epidemic gradually declining by the end of February. Before the Wuhan lockdown, the SPHCC had received its first confirmed COVID-19 patient. Distribution of the daily admission frequency was consistent with the timing of the Spring Festival holiday and accompanying large scale domestic population movement. Control measures taken in most provinces during the holiday and prior to return travel coincided with the acceleration of the Wuhan outbreak and subsequent lockdown on January 24th. Clinical data indicating that patients presented relatively early in their disease course, suggested that this relatively closed epidemiological system helped prevent additional case importation, while aggressive control measures appear to have interrupted the establishment of urban community transmission.

Although most cases presented with fever, 7% never developed fever throughout their illness. Fever was an important clue for detecting imported cases. Significant reduction of lymphopenia and CD4+T lymphopenia as well as ground-glass lesions on CT images appeared in most patients at admission, supporting the three clinical criteria i.e. fever/respiratory symptoms, leukopenia and/or lymphopenia, and typical pulmonary imaging findings, for diagnosis of suspected COVID-19 [6]. We also found that CT imaging helped classify disease severity as a larger proportion of scans from critically ill patients revealed bilateral lung involvement compared with mild cases. Data from previous studies indicating a superior sensitivity of RT-PCR in the early infection phase (0–2 days) compared with chest CT (97% vs 44%) demonstrated the importance of ensuring an adequate supply of RT-PCR services in outbreak control [11].

Abnormally elevated D-dimer levels were found in over one-third patients. Zhong et al. reported that the proportion of patients with D-dimer over 0.5 µg/ml were significantly higher in severe cases and those who met a composite endpoint i.e. admission to an intensive care unit, the use of mechanical ventilation, or death, compared with mild cases [7]. A study by Cao et al. revealed increased odds of hospital-death associated with D-dimer concentration over 1 µg/ml at admission [12]. These findings may reflect the underlying imbalance of the coagulation system triggered by infection, which warrants continued study.

Although most patients in this study presented with mild symptoms (92%), half needed oxygen therapies at least once throughout the disease course. This result was similar to other studies from Hubei province [13,14]. For example, a study in Wuhan found that a higher proportion of non-survivors received HFNO therapy, mechanical ventilation and ECMO with 19.5%, 5.2% and 0%, surviving after receiving these treatments, respectively [12]. In our study, the corresponding proportion of all cases who received these therapies were 7%, 7% and 2%, which was indicative of the relatively strong pathogenicity of SARS-CoV-2.

In chest CTs, the most typical character was ground-glass opacity observed in either or both lungs. Consistent with limited host immunity and failure to constrain the initial infection, most infiltrations appeared in the peripheral field with pleural thickening observed in 65%. Due to the general lack of specific immunity to this emerging pathogen, we observed each patient closely, and offered individualized treatment and support.

Although specific anti-SARS-CoV-2 drugs are currently unavailable, most of our patients received general antiviral agents on the first day of hospitalization. Median time to viral conversion from initiation of antiviral treatments (or admission) using pharyngeal swab, urine, faeces, and blood were 2, 2, 4, and 9 days, respectively. From disease onset to viral conversion, the median duration was 8, 8, 10, and 14 days, respectively. In contrast, the median onset-conversion duration for pharyngeal swab was 20 days in Wuhan, and the shortest time was 8 days [12]. It was unclear if general antiviral agents have a role to play in shortening the conversion duration, in part due to a shorter median onset-admission interval in Shanghai compared with Wuhan (4 days vs 11 days). Our data indicated that the median onset-conversion duration was longer in patients with severe-critical disease. There was no evidence for any benefits from antiviral treatment in terms of viral shedding when comparing mild patients using antivirals (*n* = 277) with those that did not (*n* = 22), suggesting that these drugs were not effective in shortening the infectious period. Longer viral shedding duration was detected in patients undergoing glucocorticoid therapy, indicating that glucocorticoid should be used with caution in COVID-19 patients. According to Chinese national guidelines [6] and WHO guidance [15], the routine use of systemic corticosteroids for the treatment of COVID-19 is not currently recommended. In our case series, longer duration of viral shedding was detected in blood and faeces in patients with mild disease who were using glucocorticoid. However, when limiting analyses to patients with severe-critical illness, no differences were observed according to glucocorticoid use. Dexamethasone has recently shown promise for reducing deaths among patients who required respiratory support [16]. At the time of writing, these data were yet to be peer-reviewed. Additional trials may be necessary to assess the impact of treatment with glucocorticoid.

Lymphopenia was the most common laboratory abnormality at admission, occurring in nearly 70% of cases. In Wuhan, the proportion with lymphopenia was unevenly distributed between severe and non-severe patients (96.1% versus 80.4%, Pearson Chi-Square = 22.069, *p* < 0.001) [7]. We found a lower proportion of cases presenting with lymphopenia in Shanghai compared with non-severe cases in Wuhan, (69.9% versus 80.4%, Pearson Chi-Square value = 14.301, *p* < 0.001). In addition to the shorter median onset-admission interval, this finding supported the idea that symptomatic patients received treatment earlier in Shanghai. Temporal distribution of lymphocyte counts throughout the disease course should be further studied to arrive at a more convincing answer. Lymphocyte counts less than 800/mm^3^ were found to be a strong predictor for death caused by viral pneumonia in the MuLBSTA score model [17]. Whether this model or lymphocyte counts can be used as a detection tool for early disease progression is unclear. Some patients were given thymosin to elicit an increase in lymphocytes, but the effect on prognosis requires further investigation. Consistent with earlier results, this study found that inflammatory markers, such as CRP, were elevated [7,17,18]. D-dimer elevation was also an important feature of the disease, seen in over one-third of cases. About a quarter of our patients were offered anticoagulant drugs. For patients with severe and critical conditions, positive culture results for blood and urine samples were common, indicating that concurrent infections may have adverse effects and preventive antibiotics may be beneficial in some patients as the disease progresses.

Previous reports found that the mean time of onset-admission interval in deceased patients was 6.2 days [18]. Data from 72,314 cases recorded by the Chinese Center for Disease Control and Prevention indicated an interval of 7 days between onset peak and diagnosis peak [19,20]. Data showing that the median time from onset to admission and diagnosis was 4 days suggested that the identification and treatment of cases, including isolation referrals, as observed in this relatively short time, suggested that prompt management after illness onset helped to blunt onward community transmission. In China, it was recommended that patients with mild disease also receive inpatient isolation and treatment.

Disease severity requiring oxygen, HFNO or mechanical ventilation typically emerged one week after onset. Multiple organ dysfunction required composite treatments and often occurred 2 weeks after disease onset. Hospitalization lasted 2 weeks, with 3 weeks being the typical period from onset to discharge. Therefore, 1–2 weeks after disease onset was critical period for diagnosis and treatment.

The case-fatality rate was lower (0.92%) than same day national data (3.6%) [21], and consistent with a previously reported comparison of the concurrent national case fatality rate of 3.57% and 0.85% outside Wuhan [22]. Studies have suggested that old age and comorbidities are risk factors for death [12,23]. In our cohort, 20% of patients were over 65 years old, nearly 30% had underlying disease. Case fatality rate in patients with and without glucocorticoid in the full case series was 12% and 0, respectively. As deaths only occurred in the severe-critical group, this comparison was inadequate to draw inferences on the impact of glucocorticoid use. The main cause of death was ARDS and MODS. The average hospital stay in Shanghai was 15 days was slightly longer than national average [7]. Risk factors for severe-critical disease and death will be examined in subsequent analyses.

In aggressive follow-up, there were no reported fevers or hospitalizations. With no human-to-human-transmission observed, COVID-19 patients had fully recovered two weeks after discharge.

We did not observe cases among children and pregnant women, which was consistent with studies showing a typically mild disease course among children and a lack of evidence for higher risks in pregnancy. Moreover, our research may not reflect the transmission and clinical features completely since undetected asymptomatic and mild cases may exist. Other limitations included small numbers of severe and critically ill patients for study and potential lack of representativeness of imported cases if extrapolated to urban community transmission.

## Conclusions

This retrospective study of 325 cases of COVID-19 in Shanghai found that the epidemic was imported and transmitted within clusters. The disease typically presented as a viral pneumonia involving both lungs, with half of cases requiring oxygen therapy. Time from onset to admission and the high proportion of mild illness suggested patients presented early in their disease course. The timing of Spring Festival in relation to the Wuhan lockdown helped to limit case importation, facilitate early presentation and management of symptomatic patients, and prevent onward community transmission. Moving forward, we hope to continue to deliver early and optimal care, limit community transmission, and tying the case fatality to the lower end of the known range.

## Supplementary Material

clean-supplymentary0614_SYZ_jm.doc
