# Supplementary material for: Epidemiology and clinical course of COVID-19 in Shanghai, China
Source: Emerg Microbes Infect. 2020 Jul 7;9(1):1537–45. doi: 10.1080/22221751.2020.1787103 (PMC7473125; doi:10.1080/22221751.2020.1787103)
Supplement: clean-supplymentary0614_SYZ_jm.doc [file TEMI_A_1787103_SM5071.doc]

**Table S1.** Viral shedding in mild COVID-19 cases with or without antivirals.

| **Variable** | **Value** | | | ***p-v*alue** |
| --- | --- | --- | --- | --- |
|  | **Overall**  **(n=299)** | **With antivirals**  **(n=277）** | **Without antivirals**  **(n=22）** |  |
| Time from onset to negative nucleic acid detection in pharyngeal swab—days | 8  (5-11) | 8  (5-11) | 9  (4-13.8) | 0.311 |
| Time from onset to negative nucleic acid detection in blood—days | 13  (9-17) | 13  (10-17) | 9.5  (6.8-16.5) | 0.120 |
| Time from onset to negative nucleic acid detection in urine—days | 7  (5-10) | 7  (5-10) | 7  (3.8-14) | 0.975 |
| Time from onset to negative nucleic acid detection in feces—days | 9.5  (6-13) | 10  (7-13) | 10  (3.8-17.5) | 0.654 |

Values are presented by median (interquartile range). Median values of cases prescribed with or without steroids are compared with Mann-Whitney U test.

**Table S2.** Viral shedding in COVID-19 patients with or without glucocorticoid.

| **Variable** | **Value** | | | ***p*-value** |
| --- | --- | --- | --- | --- |
|  | **Overall**  **(n=325)** | **With glucocorticoid**  **(n=50）** | **Without glucocorticoid**  **(n=275)** |  |
| Time from onset to negative nucleic acid detection in pharyngeal swab—days | 8  (5-12) | 10  (7-16) | 8  (5-11) | <0.05 |
| Time from onset to negative nucleic acid detection in blood—days | 13  (10-18) | 20.5  (12.8-26) | 13  (9-16.3) | <0.05 |
| Time from onset to negative nucleic acid detection in urine—days | 8  (5-11) | 9  (6-13) | 7  (5-10) | <0.05 |
| Time from onset to negative nucleic acid detection in feces—days | 10  (7-15) | 16  (11-20.5) | 9  (6-13) | <0.05 |
| Case fatality rate(%) | 0.92% | 12% | 0 | <0.05 |

Values are presented by median (interquartile range). Median values of cases prescribed with or without steroids are compared with Mann-Whitney U test.

**Table S3. Viral shedding in COVID-19 mild patients with or without glucocorticoid.**

| **Variable** | **Value** | | | ***p-v*alue** |
| --- | --- | --- | --- | --- |
|  | **Overall**  **（n=299）** | **with glucocorticoid**  **（n=27）** | **without glucocorticoid**  **（n=272）** |  |
| Time from onset to negative nucleic acid detection in pharyngeal swab—days | 8  (5-11) | 8  (6-11) | 8  (5-11) | 0.436 |
| Time from onset to negative nucleic acid detection in blood—days | 13  (9-17) | 18  (11-22) | 13  (9-16) | 0.003 |
| Time from onset to negative nucleic acid detection in urine—days | 7  (5-10) | 8  (6-12) | 7  (5-10) | 0.158 |
| Time from onset to negative nucleic acid detection in feces—days | 10  (7-13) | 13  (9-17) | 9  (6-13) | 0.003 |
| Case fatality rate(%) | 0 | 0 | 0 | - |

Values are presented by median (interquartile range). Median values of cases prescribed with or without steroids are compared with Mann-Whitney U test.

**Table S4. Viral shedding in COVID-19 severe-critical patients with or without glucocorticoid.**

| **Variable** | **Value** | | | ***p-v*alue** |
| --- | --- | --- | --- | --- |
|  | **Overall**  **（n=26）** | **with glucocorticoid**  **（n=23）** | **without glucocorticoid**  **（n=3）** |  |
| Time from onset to negative nucleic acid detection in pharyngeal swab—days | 14.5±7.2 | 15.4±7.24 | 8.3±0.58 | 0.170 |
| Time from onset to negative nucleic acid detection in blood—days | 23±8.5 | 22.5±8.7 | 25.7±7.5 | 0.502 |
| Time from onset to negative nucleic acid detection in urine—days | 12.5±8.7 | 13±9.3 | 9±1.7 | 0.492 |
| Time from onset to negative nucleic acid detection in feces—days | 22±8.7 | 22.3±8.9 | 20±8.2 | 0.793 |
| Case fatality rate(%) | 11.54 | 13.04 | 0 | 0.681 |

Values are presented by Mean (Standard deviation). Median values of cases prescribed with or without steroids are compared with Mann-Whitney U test.


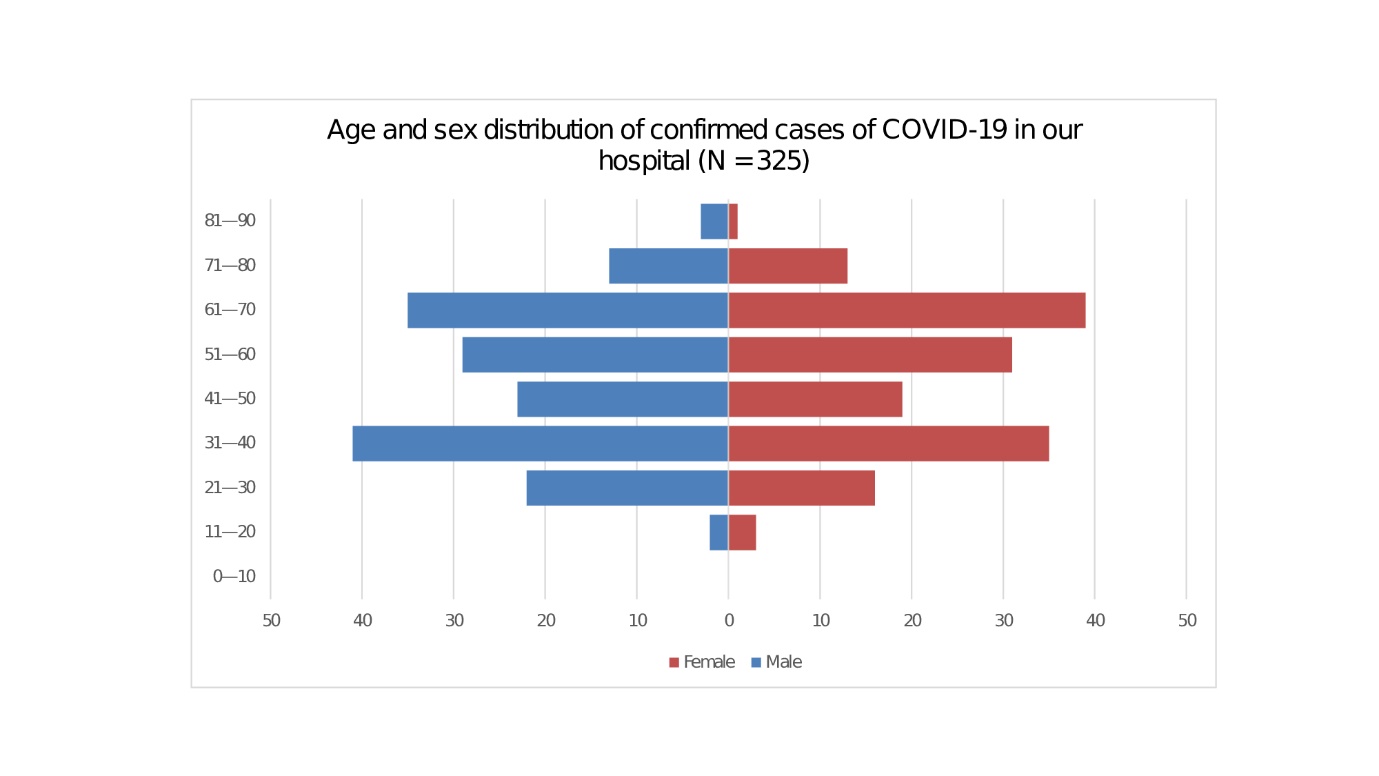
Figure S1

Age and gender distribution in confirmed COVID-19 cases admitted to Shanghai Public Health Clinical Center from January 20 to February 29, 2020.
